# Comparison of dexmedetomidine and droperidol-fentanyl for asleep-awake-asleep techniques in awake craniotomy anesthesia: a randomized controlled study

**DOI:** 10.1186/s12871-026-04117-y

**Published:** 2026-07-23

**Authors:** Xiaomei Yang, Dongdong Qu, Yiyan Ren, Yaqiu Guo, Jianpeng Wang, Jianbo Wu, Jianchun Fei, Zhigang Wang, Dejie Liu

**Affiliations:** 1 Department of Anesthesiology, Cheeloo College of Medicine, Shandong University, Jinan, Shandong 250012 China; 2 Department of Anesthesiology, Cheeloo College of Medicine, Qilu Hospital, Shandong University, Jinan, Shandong 250012 China; 3 Department of Anesthesiology, Jinan Maternity and Child Care Hospital Affiliated to Shandong First Medical University, Jinan, Shandong 250012 China; 4 Department of Anesthesiology, The First Affiliated Hospital of Shandong First Medical University & Shandong Provincial Qianfoshan Hospital, Jinan, Shandong 250014 China; 5 Department of Anesthesiology, Affiliated Hospital of Shandong University of Traditional Chinese Medicine, Jinan, Shandong 250014 China

**Keywords:** Awake craniotomy, Asleep-awake-asleep, Dexmedetomidine, Droperidol-fentanyl, Wake-up time, Eloquent cortex mapping

## Abstract

**Background:**

In this study, we aimed to compare dexmedetomidine with droperidol-fentanyl during the intraoperative awakening phase of awake craniotomy (AC), focusing on wake-up time.

**Methods:**

We conducted a single-center randomized controlled trial in China from January 2018 to December 2018. Patients aged 36–60 years, with American Society of Anesthesiologists physical status I–II, scheduled for brain glioma resection, were screened for eligibility; 61 were randomized, and 60 were included in the final analysis. The patients were randomized to either the dexmedetomidine group (intravenous loading dose of dexmedetomidine 0.5 µg·kg^− 1^ after discontinuation of propofol and remifentanil, followed by a maintenance infusion of dexmedetomidine 0.3–0.5 µg·kg^− 1^·h^− 1^) or the droperidol-fentanyl group (intravenous loading doses of fentanyl 2 µg·kg^− 1^ and droperidol 5 mg after discontinuation of propofol and remifentanil, followed by continuous maintenance infusions of fentanyl 2 µg·kg^− 1^·h^− 1^ and droperidol 0.1 mg·kg^− 1^·h^− 1^). A wake-up assessment was performed during maintenance of the assigned study regimen. The primary outcome measure was wake-up time. Secondary outcomes included the Observer’s Assessment of Alertness/Sedation (OAA/S) scale, Numerical Rating Scale (NRS), eloquent brain area mapping status, vital signs, and adverse events.

**Results:**

Baseline characteristics were comparable between the groups. Compared with the droperidol-fentanyl group, the dexmedetomidine group had lower mean arterial pressure at T4-T7, lower heart rate at T1-T4 and T7, and a shorter wake-up time, with a between-group difference of 3.5 min (95% CI, 1.95 to 5.05; *P* < 0.001). No statistically significant differences were detected in OAA/S scores at wake-up (T2), NRS scores, eloquent area mapping status, or adverse events between the groups.

**Conclusion:**

No statistically significant differences were detected in OAA/S scores at wake-up or intraoperative brain mapping performance between the two groups. The dexmedetomidine regimen was associated with a shorter measured wake-up time.

**Trial registration:**

The Chinese Clinical Trial Registry (ChiCTR1800014329) was retrospectively registered on 6 January 2018.

**Supplementary Information:**

The online version contains supplementary material available at https://doi.org/10.1186/s12871-026-04117-y.

## Background

Surgical resection of gliomas can damage functional brain areas, causing language and motor dysfunction and thereby negatively affecting patients’ postoperative quality of life. Awake craniotomy (AC) is the preferred approach for the resection of brain tumors in the eloquent cortex, which controls sensory, motor, and language functions [1]. As described in previous studies [2, 3], the two main benefits of AC are the maximization of safe tumor resection and preservation of neurological function. As a result, AC has gradually become a standard protocol for brain glioma resection. Anesthesia techniques such as the “asleep-awake” or the asleep-awake-asleep (AAA) anesthesia technique, which typically consist of general anesthesia and airway management, have been successfully implemented during AC [4]. General anesthesia used for the AAA technique is commonly facilitated by anesthetic agents, such as remifentanil, propofol infusions, and muscle relaxants [5, 6]. The laryngeal mask airway (LMA) is typically used and usually no or minimal airway manipulation is required during AAA. General anesthetic infusions, such as propofol and remifentanil, are discontinued approximately 15 min before brain functional testing, after which patients are awakened as spontaneous ventilation returns, and the LMA is removed once they respond to commands. Anesthesia is re-induced at the end of neurological testing for the rest of the surgery.

Dexmedetomidine (DEX) provides sympatholytic, sedative, analgesic, and arousal properties with minimal respiratory depression [7, 8] and may be useful for general anesthesia in neurosurgical procedures. Bekker [9] first provided a case report of the use of DEX during AC in 2001. Several randomized controlled trials have investigated perioperative DEX administration; however, the conclusions have varied [10–13]. A recent study reported that a loading dose of DEX during anesthesia facilitated the effect of analgesic medicine, reduced post-operative vomiting and restlessness, and increased patient satisfaction with anesthesia [4].

The combination of droperidol and fentanyl (droperidol-fentanyl), a form of neuroleptanalgesia, has been used in clinical anesthesia since the 1960s [14]. Fentanyl is a widely used synthetic opioid and a highly selective µ-opioid receptor agonist [15]. Droperidol is a strong neuroplegic dopamine receptor antagonist that induces psychic apathy, kinetic reflex restraint, and nerve truncation with steadiness of the autonomic nervous system. Droperidol-fentanyl provides neurovegetative stability and is used in neurosurgical procedures. The pharmacological characteristics of DEX and droperidol-fentanyl in sedation, analgesia, inhibition of post-operative nausea and vomiting (PONV), and non-inhibition of respiration make them suitable for additional sedation during arousal anesthesia. Previous studies of anesthesia for AC have mainly focused on the feasibility of different anesthetic techniques, such as monitored anesthesia care versus the AAA technique [4], or on the use of DEX in comparison with propofol- or remifentanil-based regimens [16–18]. These studies suggest that DEX can be used during AC, but the reported effects on wake-up quality, hemodynamics, respiratory safety, and intraoperative testing have not been fully consistent. To date, comparative studies of DEX and droperidol-fentanyl in arousal anesthesia have rarely been reported.

Based on these considerations, this randomized controlled study was designed to compare DEX with droperidol-fentanyl as the awake-phase regimen for AAA anesthesia in patients undergoing glioma resection, with wake-up time as the primary outcome and sedation level at wake-up, pain, cooperation during eloquent cortex mapping, vital signs, and adverse events as secondary outcomes.

## Methods

### Study design

This single-center, randomized controlled trial was approved by the local ethics committee of Qilu Hospital, Cheeloo College of Medicine, Shandong University, Jinan, China, on February 25, 2017 (The reference number KYLL-2017-077, Chairperson Professor Xiaoyang Chen) and the Chinese Clinical Trial Registry at chictr.org.cn (ChiCTR1800014329). The first participant was enrolled on January 2, 2018; therefore, the trial was registered retrospectively. The registry entry was not fully consistent with the final manuscript with respect to some design elements, including outcome designation, sample size, and eligibility criteria. To improve transparency, a concise registry-to-manuscript reconciliation is provided in Supplementary Table 1. This report describes the methods used in this study. Written informed consent was obtained from all study participants. This randomized controlled trial was conducted in accordance with the Consolidated Standards of Reporting Trials (CONSORT) guidelines.

### Patient recruitment

We consecutively enrolled patients aged 36–60 years, classified as American Society of Anesthesiologists physical status I–II, with brain gliomas and no history of surgery or chemoradiation between January 2018 and December 2018. All patients underwent complete psychological, neurological, and neuroradiological evaluations prior to surgery. Eligible patients were screened according to the predefined inclusion and exclusion criteria. Patients with severe cognitive or psychologic disturbances and those with difficulty in communication, regularly taking α2 agonists or benzodiazepines, with known alcohol or substance abuse, allergies to the drugs being used, unable to understand the Numerical Rating Scale (NRS), severe obesity, difficult airways, or declined to participate were excluded [19, 20]. Preoperative 12-lead ECG findings were reviewed during routine preoperative assessment.

### Anesthesia induction and maintenance.

After establishment of the peripheral venous catheter and comfortable positioning on the operating table, continuous electrocardiography (lead II), oxygen saturation, HR, and invasive BP were monitored from anesthesia induction until the end of the operation. Anesthesia management was performed using predefined protocols for both groups. Each patient received midazolam 0.05 mg·kg^−1^, remifentanil 1.5 µg·kg^−1^, propofol 2 mg·kg^−1^, and rocuronium 0.6 mg·kg^−1^ for induction of anesthesia.

The LMA was inserted after ventilation with a mask for 3 min. End-tidal carbon dioxide (EtCO2) was monitored to determine the respiratory rate (RR) and to maintain EtCO2 between 35 and 45 mmHg. Propofol was administered by TCI using the Marsh model, with a target concentration of 2.5 µg·mL^−1^, and remifentanil was infused at 0.1 µg·kg^−1^·min^−1^ throughout the procedure.

### Scalp nerve block

At the beginning of the surgery, scalp nerve blocks were performed using 6–10 mL of bupivacaine (0.375%). An 8-point scalp nerve block was marked and injected with bupivacaine (0.375%) without epinephrine (20–25 mL) [21], and 2–3 mL of local infiltration was used at each injection site [22]. The participants were secured in a Mayfield headholder. A 3-pin head holder was placed on the patient under local anesthesia with lidocaine 2% (5–10 mL). Additional muscle penetration and local anesthesia of the dura (2% lidocaine) were administered when necessary.

### Interventions

Before surgery, eligible patients were randomized equally to receive either DEX (D group) or droperidol-fentanyl (D-F group) infusions. Patients in the two groups underwent general anesthesia induction, and an LMA was used to control ventilation. Approximately 15 min prior to the desired electrophysiological detection [23], the surgeons informed the anesthetists, and the infusions of propofol and remifentanil were stopped. The D group received an intravenous loading dose of 0.5 µg·kg^− 1^ DEX over 10 min, then maintained with a continuous infusion rate of 0.3–0.5 µg·kg^− 1^·h^− 1^. The D-F group received an intravenous injection of fentanyl 2 µg·kg^− 1^ and droperidol 5 mg as a loading dose, maintained by continuous infusion of fentanyl 2 µg·kg^− 1^·h^− 1^ and droperidol 0.1 mg·kg^− 1^·h^− 1^. These maintenance infusions were continued during wake-up assessment, cortical mapping, and tumor resection until completion of the awake phase unless they had to be discontinued because of persistent hemodynamic instability. Motor, sensory, and language functions were assessed during cortical mapping. At any time during the awake phase, if the patient complained of discomfort or pain, the infusion rate in either group was adjusted to within the predefined maintenance range, as clinically indicated.

### Surgical procedures and anesthesia awareness

Before electrophysiological monitoring, patients were required to meet the predefined wake-up criteria and considered ready for intraoperative testing. Readiness for intraoperative testing was defined as the fulfillment of the predefined wake-up criteria. A wake-up assessment was performed during maintenance of the assigned study regimen. Wake-up time was measured from the discontinuation of propofol and remifentanil until all predefined wake-up criteria were fulfilled. Neuromuscular function was monitored using train-of-four (TOF) stimulation. Immediately after discontinuation of propofol and remifentanil, neuromuscular blockade was routinely antagonized with intravenous neostigmine 0.03 mg·kg^−1^, up to a maximum dose of 2 mg according to institutional practice. The LMA was removed only after adequate clinical recovery of muscle strength and confirmation of a TOF ratio ≥ 0.9, before intraoperative testing. Oxygen was then administered via a face mask. According to language and motor functional area monitoring, the following tasks were performed: (1) a counting task, in which the patient was asked to count from 0 to 100 and repeat the sequence; (2) a naming task, in which 10 picture cards of common animals were presented, and the patient was familiarized with the animal names before surgery; and (3) a motor task, in which the patient was asked to relax the whole body, fully expose the limbs contralateral to the lesion, and move the hands and legs on command.

When blood pressure fluctuated by more than 20% from baseline, metaraminol 0.1–0.2 mg or urapidil 10–15 mg was administered as appropriate. If blood pressure remained > 20% below the baseline despite vasoactive treatment, the assigned study regimen was discontinued. Atropine 0.5 mg was administered when the heart rate decreased to ≤ 45 beats·min^−1^, and 10–20 mg esmolol was administered when the heart rate increased to 100 beats·min^−1^. Continuous verbal reassurance was provided throughout the awake phase to maintain the patients’ trust and cooperation.

### Reinduction of general anesthesia

General anesthesia was reinduced after completion of tumor resection and neurophysiological testing. Each patient received oxygen (3–5 L/min) via face mask and was administered remifentanil 0.15 µg·kg^−1^, propofol 2 mg·kg^−1^, and rocuronium 0.6 mg·kg^− 1^ for reinduction of anesthesia. After consciousness disappeared, the LMA was reimplanted and confirmed to be in the correct position, with normal airway pressure and no air leakage after mechanical ventilation. After surgery, the patients were sent to the post-anesthesia care unit for 1 h of monitoring. Finally, they were returned to the ward when their voluntary breathing and muscle strength had recovered.

### Primary outcome measure

The primary outcome was wake-up time, defined as the interval from the discontinuation of propofol and remifentanil infusions to the first fulfillment of the predefined wake-up criteria. The wake-up criteria were the ability to open the eyes on commands, obey simple verbal commands, and perform purposeful voluntary movements of the fingers and toes [24].

Wake-up assessments were conducted using a standardized protocol by trained anesthesia personnel who were not involved in the intraoperative drug administration. Assessments were performed at fixed 15-second intervals. At each assessment, standardized verbal commands were delivered first. If no purposeful response was observed, gentle shoulder tapping was applied as the secondary stimulus. The standardized verbal commands included eye opening, hand squeezing, and voluntary movements of the fingers and toes. Identical wake-up criteria and command sequences were used for both groups throughout the study. The wake-up time was recorded when all predefined wake-up criteria were satisfied.

### Secondary outcome measures

Secondary outcome measures included the following: (1) eloquent brain area mapping status to assess the ability of the participants to cooperate and accomplish the mapping, which was assessed using language (1: blurred; 2: fuzzy; 3: medium clarity; 4: clear), ability to follow commands (1: dullest; 2: duller; 3: more accurate; 4: most accurate), pain (1: severe pain; 2: moderate; 3: mild; 4: no pain), and intracranial pressure (1: swelling; 2: significantly elevated; 3: slightly elevated; 4: normal). The total eloquent brain area mapping status was divided into three grades: (excellent, 16 points; good, 12–15 points; and poor, < 12 points). (2) HR, mean arterial pressure (MAP), and SpO2 were recorded at eight successive time points: baseline (T0), immediately after the discontinuation of propofol and remifentanil infusions (T1), wake-up (T2), and 5 min (T3), 10 min (T4), 15 min (T5), 30 min (T6), and 60 min (T7) after waking. (3) A 10-point NRS was used to measure pain during the wake-up period, and the patients were asked to evaluate the level of pain (0 = no pain; 1–3 = mild pain; 4–6 = moderate pain; 7–10 = severe pain). (4) Sedation level was assessed at wake-up (T2) using the modified Observer’s Assessment of Alertness/Sedation (OAA/S) scale (1 = response only to noxious stimuli, 2 = response only to mild physical stimulation, 3 = response to repeated or loud verbal stimulation, 4 = delayed response to verbal stimulation, and 5 = prompt response to verbal stimulation). (5) Hemodynamic instability, including cardiac arrhythmia, arterial hypotension, or hypertension, and respiratory depression events, including oxygen desaturation, airway obstruction, hypoventilation, and apnea, were defined as adverse events when treatment intervention was required, including pharmacologic treatment for hemodynamic events or airway support for respiratory events. Other complications, such as postoperative PONV, shivering, agitation, failure to provide appropriate analgesia, and all post-operative complications, were defined as adverse events.

### Sample size and randomization

The sample size was calculated based on the primary outcome of wake-up time. Based on our preliminary data, the mean wake-up time for droperidol-fentanyl was estimated to be 16.5 ± 4.2 min. We considered a reduction of 20% in the dexmedetomidine group (Δ = 3.3 min) to be clinically significant. Using an independent two-sample t-test with a two-sided significance level (α) of 0.05 and a power (1-β) of 0.80, the calculation (via G*Power 3.1.9.7 software) indicated that 27 patients per group were required. Anticipating a potential dropout or protocol violation rate of approximately 10%, 30 patients per group were enrolled, resulting in a total sample size of 60 patients. Eligible patients were randomly assigned in a 1:1 ratio using a computer-generated random sequence prepared by an investigator who was not involved in patient enrollment or outcome assessment. Group allocation was disclosed to the anesthesia personnel responsible for drug administration only after patient inclusion; the outcome assessors were not involved in the randomization process.

### Statistical analysis

Statistical analyses were performed using SPSS (version 20.0; IBM Corp., Armonk, NY, USA). Continuous variables were compared between groups using independent-sample t-tests when approximately normally distributed; otherwise, the Mann–Whitney U test was used. For the primary outcome, between-group mean differences and 95% confidence intervals were calculated. Pre-specified between-group comparisons of HR, MAP, and SpO_2_ were performed separately at each recorded time point. These analyses were not adjusted for multiple comparisons and should therefore be interpreted as exploratory. Ordinal outcomes, including the OAA/S score at wake-up (T2), NRS score, and eloquent cortex mapping status, were compared between the groups using the Mann–Whitney U test. Categorical variables were analyzed using the χ^²^ test or Fisher’s exact test, as appropriate. Statistical significance was defined as a two-sided *P*-value of < 0.05.

## Results

### Patient characteristics

**Fig. 1:**
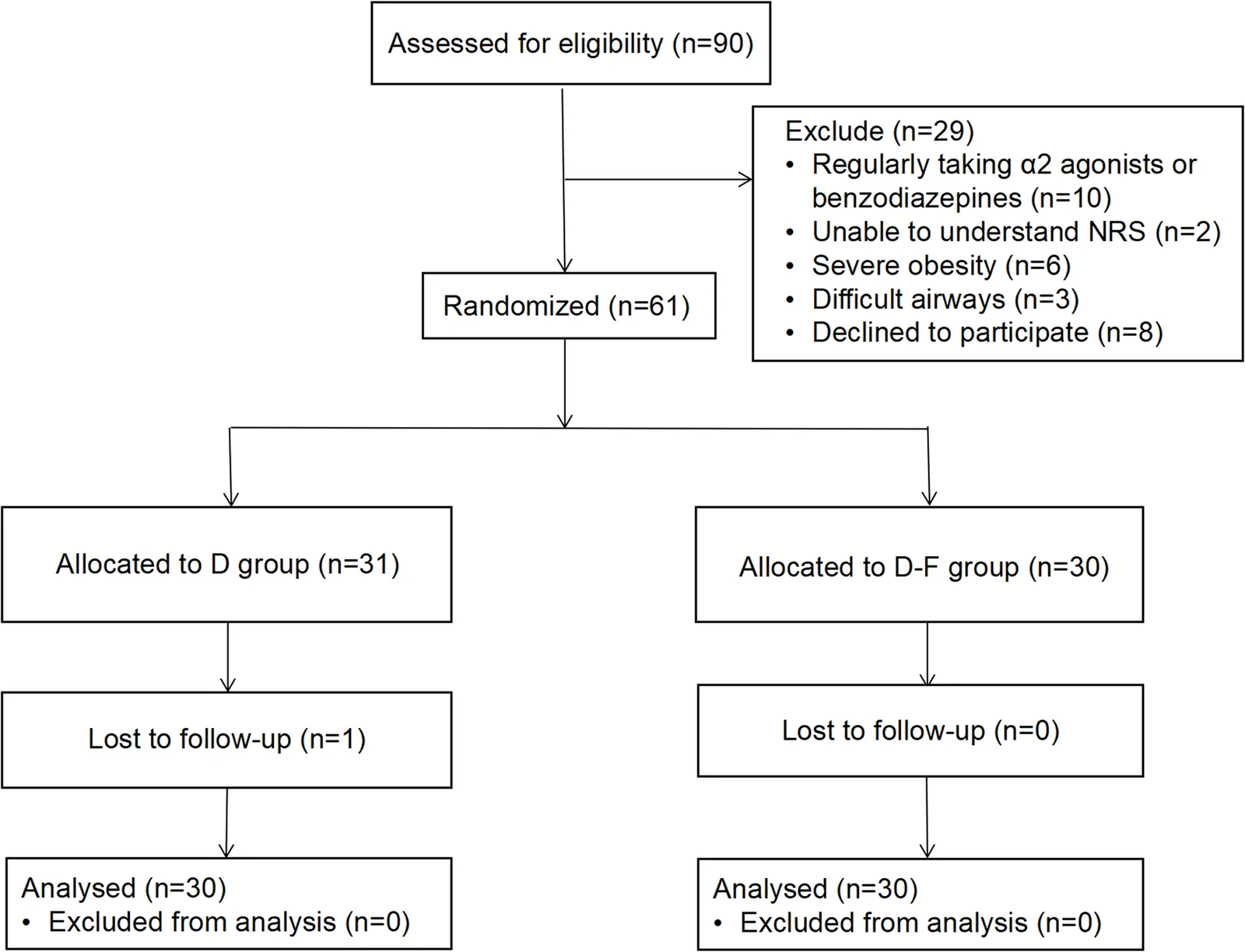
CONSORT flow diagram. Ninety patients were assessed for eligibility, 61 were randomized, and 60 were included in the final analysis. D group, dexmedetomidine group; D-F group, droperidol-fentanyl group

Between January and December 2018, 90 patients were assessed for eligibility (Fig. 1). Of these, 29 were excluded before randomization because they were regularly taking α2 agonists or benzodiazepines (n = 10), were unable to understand the NRS (n = 2), had severe obesity (n = 6), difficult airways (n = 3), or declined to participate (n = 8). The remaining 61 patients were randomized to either the D group (n = 31) or D-F group (n = 30). One participant in group D was lost to follow-up, leaving 30 patients in each group for analysis. Baseline characteristics are shown in Table 1. Sex, age, height, weight, and BMI were comparable between the two groups.

**Table 1:** Characteristics of the two groups

Group	D group (*n* = 30)	D-F group (*n* = 30)
Sex (M/F)	13/17	11/19
Age (years)	49.23 ± 9.13	52.57 ± 6.31
Height (cm)	161.73 ± 5.53	162.87 ± 6.18
Weight (kg)	68.60 ± 10.24	70.43 ± 9.13
BMI (kg/m^2^)	26.24 ± 3.86	26.60 ± 3.60

Data are expressed as mean ± SD, except for Sex (M/F)

*D* dexmedetomidine group *D-F* group, droperidol-fentanyl group, *BMI* body mass index,  *F* female, *M* male

**Fig. 2:**
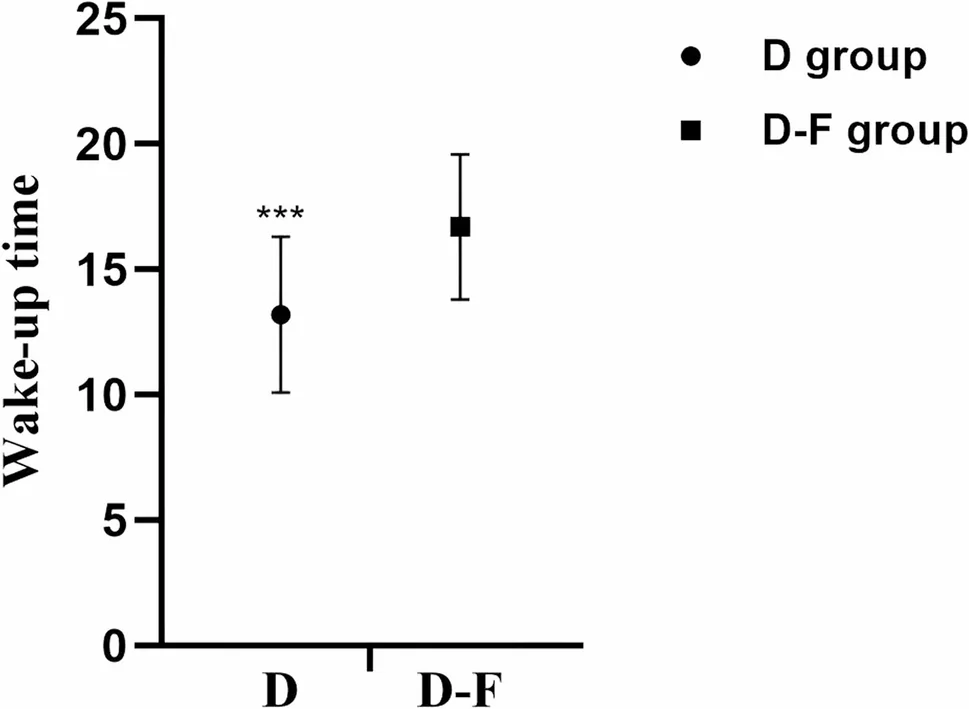
Comparison of wake-up time between the two groups. Data are presented as mean (SD). The between-group difference was 3.5 min (95% CI, 1.95 to 5.05). ***P < 0.001. D group, dexmedetomidine group; D-F group, droperidol-fentanyl group

### Wake-up time

The wake-up time was successfully recorded for all patients. It was shorter in the D group than in the D-F group (13.2 ± 3.1 min vs. 16.7 ± 2.9 min), with a between-group difference of 3.5 min (95% CI, 1.95 to 5.05; P < 0.001; Fig. 2).

### Vital signs (HR, MAP, and SpO_2_) during the procedure

**Fig. 3:**
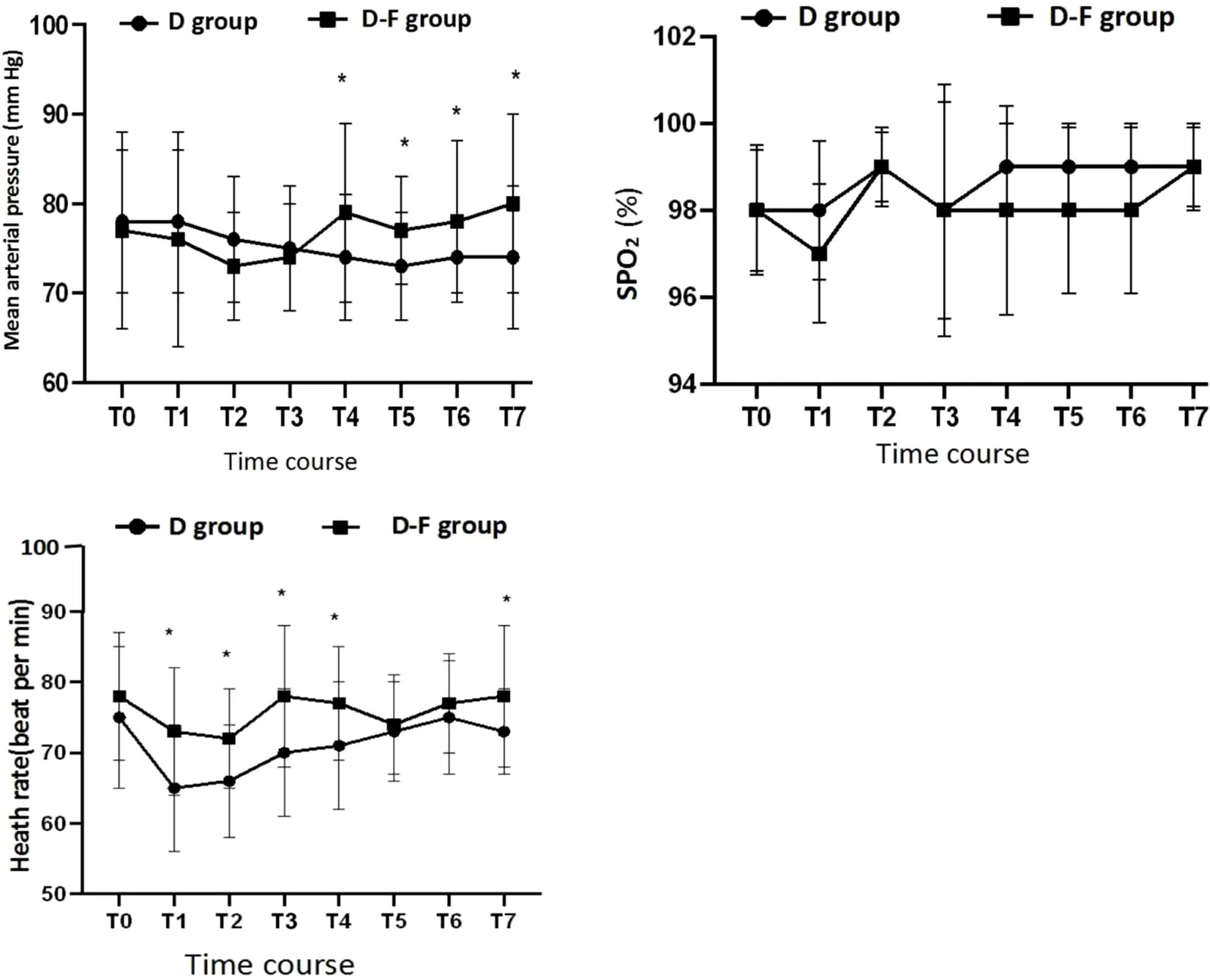
Comparison of vital signs (MAP, SpO2, and HR) at consecutive time points (T0–T7). **(A)** Mean arterial pressure (MAP, mmHg), **(B)** peripheral oxygen saturation (SpO2, %), and **(C)** heart rate (HR, beats·min-1). Data are presented as mean (SD). D group, dexmedetomidine group; D-F group, droperidol-fentanyl group. T0, baseline; T1, immediately after discontinuation of propofol and remifentanil infusions; T2, wake-up; T3–T7, 5, 10, 15, 30, and 60 min after wake-up, respectively. *P < 0.05

At the pre-specified time-point comparisons, the HR was lower in group D than in groups D–F at T1–T4 and T7 (*P* = 0.002, 0.003, 0.002, 0.005, and 0.017, respectively; Fig. 3). The MAP was also lower in group D at T4–T7 (*P* = 0.049, 0.019, 0.033, and 0.007, respectively). No significant between-group differences in SpO_2_ were observed at any time point (*P* > 0.05; Fig. 3). These hemodynamic findings were based on prespecified between-group comparisons at individual time points and were not adjusted for multiple comparisons. They should therefore be interpreted as exploratory secondary findings.

### Eloquent brain areas mapping status, OAA/S, and NRS

As summarized in Table 2, no statistically significant between-group differences were detected in eloquent brain area mapping status (*P* = 0.635), OAA/S scores at wake-up (T2) (*P* = 0.606), or NRS scores (*P* = 0.929).

**Table 2:** Comparison of eloquent brain areas mapping status, OAA/S score at wake-up (T2), and NRS

Outcome	Category	D group (*n* = 30)	D-F group (*n* = 30)	*P* value
OAA/S				0.606
	5	22 (73%)	20 (67%)	
	4	6 (20%)	8 (27%)	
	3	2 (7%)	2 (7%)	
	2	0 (0%)	0 (0%)	
	1	0 (0%)	0 (0%)	
NRS				0.929
	No pain	20 (67%)	20 (67%)	
	Mild pain	6 (20%)	7 (23%)	
	Moderate pain	4 (13%)	3 (10%)	
	Severe pain	0 (0%)	0 (0%)	
Mapping status				0.635
	Excellent	8 (27%)	5 (17%)	
	Good	18 (60%)	20 (67%)	
	Poor	4 (13%)	5 (17%)	

OAA/S, Modified Observer’s Assessment of Alertness/Sedation (OAA/S) scale;

*NRS* Numerical Rating Scale for pain, *D* dexmedetomidine group, *D-F* group, droperidol-fentanyl group

### Adverse events

Adverse events are summarized in Table 3. No statistically significant between-group differences were observed in PONV (3/30 [10.0%] vs. 4/30 [13.3%]; P = 1.000), intraoperative desaturation (2/30 [6.7%] vs. 5/30 [16.7%]; P = 0.424), hemodynamic instability (1/30 [3.3%] vs. 1/30 [3.3%]; P = 1.000), shivering (2/30 [6.7%] vs. 1/30 [3.3%]; P = 1.000), or agitation (3/30 [10.0%] vs. 2/30 [6.7%]; P = 1.000). No treatment-requiring arrhythmias were documented in either group.

**Table 3:** Incidence of adverse events

Adverse events	D group (*n* = 30)	D-F group (*n* = 30)	*P* value
PONV, n (%)	3 (10.0)	4 (13.3)	1
Episodes of intraoperative desaturation, n (%)	2 (6.7)	5 (16.7)	0.424
Hemodynamic instability, n (%)	1 (3.3)	1 (3.3)	1
Shivering, n (%)	2 (6.7)	1 (3.3)	1
Agitation, n (%)	3 (10.0)	2 (6.7)	1

Data are expressed as numbers (%)

*PONV* postoperative nausea and vomiting, *D* dexmedetomidine group, *D-F* group, droperidol-fentanyl group

## Discussion

AC has been increasingly performed over the past decades [25–28]. It has mainly been used for the resection of brain tumors. AC enables surgeons to monitor eloquent cortical functions, including language, motor, and sensory functions, during surgery and may thereby reduce the risk of new or worsened postoperative neurological deficits [3]. An intra-operative wake up test requires an anesthesia scheme that allows rapid emergence to a level of consciousness where a response to orders can be achieved [29, 30]. In this study, we found that either DEX or droperidol-fentanyl could be used to achieve an appropriate intraoperative awake state during brain glioma resection surgery. Successive studies assessing the effects of DEX on the capacity to perform intra-operative neurological testing have reported inconsistent results [31, 32]. In this study, the wake-up time was shorter in the D group than in the D-F group. This finding may be related to the arousable sedative profile of dexmedetomidine, which facilitates the transition from sedation to wakefulness during intraoperative awakening. However, because the loading regimens differed in timing and pharmacokinetic profile during the T1-to-T2 measurement window, the observed between-group difference may also have been influenced by regimen-related differences during early emergence. Therefore, our findings support an association between the assigned awake-phase regimen and wake-up time within the study design used. Nevertheless, the aim of the present study was to compare the overall clinical performance of the awake-phase regimens rather than to isolate the relative contributions of dexmedetomidine pharmacology and management-related factors to the primary outcome. In addition, although blinding of anesthesia personnel was not feasible, wake-up time was assessed using predefined criteria and a standardized protocol, which may have reduced, but could not eliminate, the risk of observer-related bias.

The initial step of head-pin insertion can be highly stimulating and painful, and a single anesthetic technique may not provide sufficient analgesia throughout AC, therefore, local anesthesia is necessary. Local anesthesia was accomplished through ‘ring block’ penetration of the cut spot with bupivacaine in patients undergoing AC and afforded conscious analgesia [22]. Previous studies have shown that OAA/S is a useful tool for assessing sedation during dexmedetomidine administration [33]. DEX has been described in pediatric awake craniotomy in case reports [34]. Moreover, several studies have reported an association between DEX and fentanyl, remifentanil, propofol, nitrous oxide or sevoflurane [9, 35]. However, the use of DEX alone has rarely been reported. In this study, the OAA/S scores assessed at wake-up (T2) did not differ significantly between the two groups. However, OAA/S was measured only at this time point, and this finding should not be interpreted as demonstrating sedation comparability throughout the entire awake phase. Similarly, no significant between-group differences were observed in NRS scores or intraoperative brain mapping status. The analgesic-sparing effect of DEX may be related to central α2-adrenoceptor-mediated modulation of nociceptive transmission. As an adrenergic receptor agonist, DEX can act on densely packed areas of the central nervous system to regulate arousal and wakefulness [36].

It is well known that reductions in MAP and HR are the most common cardiovascular effects of DEX [37, 38]. It can also cause an early decline in HR. Clinically important episodes of bradycardia and hypotension have been associated with DEX and may require medical intervention in 3% and 10% of patients, respectively [38]. Low-dose infusion of DEX significantly reduced MAP during waking in previous studies [16]. Droperidol did not inhibit respiration or myocardial contractility but had mild α-epinephrine receptor-blocking action, which may lead to a slight drop in BP during rapid intravenous administration in patients with hypovolemia. In the present study, DEX was associated with a lower HR at T1–T4 and T7 and a lower MAP at T4–T7 than droperidol-fentanyl. These differences were observed at prespecified intraoperative time points and should be interpreted as exploratory secondary findings. In addition, the reasonably low occurrence rate of adverse hemodynamic events during conscious sedation for AC found in both the D and D-F groups was consistent with the findings of previous studies [13, 17]. From a practical perspective, dexmedetomidine may be a reasonable option for the awake phase of AC when a shorter wake-up time is clinically desirable, with no statistically significant differences detected in wake-up sedation or brain mapping performance in this study.

The main safety points associated with conscious sedation in non-intubated patients include airway compromise, hypoventilation, and oxygen desaturation. A higher incidence of intraoperative desaturation was observed in the D-F group, although the between-group difference was not statistically significant. This observation may be partly related to the relatively high fentanyl infusion rate used in the D-F group during the awake phase. Unlike opioids such as fentanyl and remifentanil, droperidol itself does not directly inhibit the respiratory center. Changes in chest wall compliance may reduce tidal volume and breathing frequency. Conscious sedation with DEX has been reported to be feasible in high-risk patients requiring careful airway management and is associated with a low requirement for active airway rescue [18, 22]. DEX alone is generally associated with limited respiratory depression. In this study, the incidence of intra- and post-operative agitation or other adverse events did not differ between the groups. PONV could be an urgent issue in patients undergoing complicated neurosurgical procedures such as AC. A previous study found that the use of DEX reduced the frequency and severity of acute post-operative nausea in highly sensitive patients before and after surgery [39]. DEX reduced PONV in pediatric patients undergoing strabismus surgery [40] and was associated with a large reduction in the relative risk of PONV compared to balanced anesthesia [41]. In contrast, DEX was not associated with a reduction in PONV in the morbidly obese patients [42]. Droperidol mainly blocks the dopamine receptors. A previous study reported that intravenous droperidol effectively decreases PONV after general anesthesia [43]. In our study, no difference was observed in the incidence of PONV among participants.

This study had several limitations. First, the relatively small sample size and single-center design may limit the generalizability of the findings. Second, although wake-up time was assessed using predefined criteria and a standardized protocol, complete blinding of awake-phase assessment was not feasible. Third, the observed difference in wake-up time may have been influenced by regimen-related pharmacokinetic and timing differences during the T1-to-T2 interval, as well as by unreported peri-emergence and surgical factors, including prior propofol/remifentanil exposure, rescue interventions, tumor-related factors, surgical duration, and maintenance EtCO₂. Fourth, the droperidol-fentanyl regimen involved a higher cumulative droperidol exposure than contemporary antiemetic dosing and should be interpreted in light of the FDA boxed warning. Although preoperative ECG findings were reviewed and continuous intraoperative ECG monitoring was performed, serial QTc intervals were not prospectively collected; therefore, QTc-related safety could not be definitively assessed. Fifth, the fentanyl infusion rate used in the D-F group was relatively high for awake-phase sedation and may have contributed to the numerically higher incidence of intraoperative desaturation. Finally, repeated hemodynamic measurements were analyzed at prespecified time points without formal longitudinal modeling or adjustment for multiple comparisons, and postoperative neurological deficits, extent of resection, and post-anesthesia care unit outcomes were not systematically assessed. Therefore, the present findings should be interpreted primarily as intraoperative procedural outcomes in patients undergoing AC for tumor resection, rather than evidence of broader postoperative or neurological benefit.

## Conclusions

In conclusion, no statistically significant differences were detected in OAA/S scores at wake-up or intraoperative brain mapping performance between the DEX and D-F groups, whereas the DEX regimen was associated with a shorter measured wake-up time. 

## Supplementary Information

Supplementary Material 1.

## Data Availability

The datasets generated and/or analyzed in the current study are available from the corresponding author upon reasonable request.

## Abbreviations

Abbreviation: Full Form
AAA: Asleep-awake-asleep
AC: Awake craniotomy
ASA: American society of anesthesiologists (physical status)
BMI: Body mass index
CI: Confidence interval
CONSORT: Consolidated standards of reporting trials
D group: Dexmedetomidine group
DEX: Dexmedetomidine
D-F group: Droperidol-fentanyl group
EtCO_2_: End-tidal carbon dioxide
F: Female
HR: Heart rate
TOF: Train-of-four
LMA: Laryngeal mask airway
M: Male
MAP: Mean arterial pressure
NRS: Numerical rating scale
OAA/S: Observer’s assessment of alertness/sedation
PONV: Postoperative nausea and vomiting
RR: Respiratory rate
SD: Standard deviation
SpO_2_: Peripheral capillary oxygen saturation
T0: Baseline (time point)
T1: Immediately after discontinuation of propofol and remifentanil infusions
T2: Wake-up
T3: 5 min after waking
T4: 10 min after waking
T5: 15 min after waking
T6: 30 min after waking
T7: 60 min after waking
TCI: Target controlled infusion
