# Supplementary material for: Comparison of dexmedetomidine and droperidol-fentanyl for asleep-awake-asleep techniques in awake craniotomy anesthesia: a randomized controlled study
Source: BMC Anesthesiol. 2026 Jul 23;26:626. doi: 10.1186/s12871-026-04117-y (PMC13679275; doi:10.1186/s12871-026-04117-y)
Supplement: Supplementary file 1 [file 12871_2026_4117_MOESM1_ESM.docx]

**Supplementary Table 1.** Concise registry-to-manuscript reconciliation

| **Item** | **Registry entry** | **Revised manuscript** |
| --- | --- | --- |
| Trial registration timing | Registered on 6 January 2018; first participant enrolled on 2 January 2018. | The trial is described as a single-center randomized controlled trial with retrospective registration. |
| Primary outcome designation | OAA/S score and intraoperative MAP. | Primary outcome: wake-up time;  OAA/S score and vital signs are reported as secondary outcomes. |
| Sample size | Target sample size: 40 participants, with 20 participants per group. | 61 patients were randomized, and 60 were included in the final analysis. The sample-size calculation was based on wake-up time. |
| Eligibility criteria | Age 40–65 years; ASA physical status II–III. | Age 36–60 years; ASA physical status I–II; patients with brain glioma and no history of surgery or chemoradiation. |

Abbreviations: ASA, American Society of Anesthesiologists physical status; MAP, mean arterial pressure; OAA/S, Observer’s Assessment of Alertness/Sedation
